# Tuning Dry and Wet Adhesion with a Branched Supramolecular Polymer Solution

**DOI:** 10.1002/smsc.202300044

**Published:** 2023-06-11

**Authors:** Huan Yu, Runlin Zhang, Yong-Guang Jia, Yunhua Chen, Xuetao Shi

**Affiliations:** ^1^ School of Materials Science and Engineering South China University of Technology Guangzhou 510640 China; ^2^ National Engineering Research Centre for Tissue Restoration and Reconstruction South China University of Technology Guangzhou 510006 China; ^3^ Key Laboratory of Biomedical Engineering of Guangdong Province, and Innovation Center for Tissue Restoration and Reconstruction South China University of Technology Guangzhou 510006 China; ^4^ Key Laboratory of Biomedical Materials and Engineering of the Ministry of Education South China University of Technology Guangzhou 510006 China

**Keywords:** branched supramolecular polymers, dry adhesion, hydrogels, hydrogen bonding, wet adhesion

## Abstract

Endowing adhesives with both dry and wet adhesion to surfaces from hard substrates to soft biological tissues is challenging. Herein, a branched supramolecular polymer is introduced in dry‐ and wet‐crosslinking forms to adaptively achieve dry and wet interfacial adhesion. The branched supramolecular polymer (PAMU) with multiple pendant functional moieties is prepared by one‐pot reversible addition‐fragmentation chain transfer polymerization. The PAMU polymer solution can firmly adhere to glass sheets after the water evaporates. The aqueous solution of PAMU polymer with residual double bonds can be further polymerized to form a wet crosslinking network. The hydrogen bonding moieties endow the supramolecular network with self‐healing properties, while the in situ polymerization of the PAMU polymer leads to stable adhesion to wet tissues. This study provides a promising approach to develop supramolecular adhesives for versatile adhesion applications.

## Graphical abstract

A branched supramolecular polymer (PAMU) with multiple pendant functional moieties is prepared by one‐pot RAFT polymerization. The PAMU polymer solution demonstrates strong dry adhesion after water evaporation. The aqueous solution can be further turned into a crosslinking hydrogel due to the presence of residual double bonds in PAMU, leading to stable wet adhesion to biological tissues.

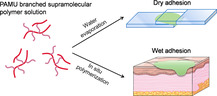

## 1 Introduction

Strong adhesion to various surfaces has a pivotal role in daily life and industrial applications, such as the manufacture of archaeological restoration,^[^ ^1^ ^]^ electronic component welding,^[^ ^2^, ^3^ ^]^ wall‐climbing robots,^[^ ^4^, ^5^ ^]^ building repair,^[^ ^6^ ^]^ and wound healing.^[^ ^7^, ^8^, ^9^, ^10^ ^]^ At present, there are many available adhesives from nature to synthetic polymers. For example, a gecko‐inspired tape has good self‐cleaning and repeated adhesion properties by van der Waals forces and capillary actions.^[^ ^11^ ^]^ The adhesive with a mushroom‐shaped structure possesses a certain suction effect when tested in vacuum.^[^ ^12^ ^]^ The poly(2‐hydroxyethyl methacrylate) (PHEMA) superglue demonstrates strong adhesion with a working mechanism similar to that of the snail epiphragm.^[^ ^13^ ^]^ Unfortunately, the application conditions of these adhesives are actually limited. Their biomimetic design only enables robust adhesion on dry surfaces. The adhesion strength would be significantly deteriorated under wet conditions.

To this end, great attention has been paid to developing wet adhesives. Adhesion in wet conditions has a wide range of applications in biomedical fields such as tissue engineering^[^ ^14^, ^15^, ^16^, ^17^ ^]^ and clinical medicine.^[^ ^18^, ^19^, ^20^ ^]^ In recent years, bioadhesives have partially replaced surgical sutures in the clinic, which can control bleeding and prevent gas or fluid leakage. It also serves as a wound dressing to rapidly treat wounds and promote tissue regeneration.^[^ ^21^, ^22^ ^]^ Inspired by nature, several prior studies have revealed that mimicking creatures such as mussels,^[^ ^23^, ^24^ ^]^ spiders,^[^ ^14^, ^25^ ^]^ and ivies^[^ ^26^, ^27^ ^]^ that are capable of fast‐forming strong adhesion in a wet environment is effective. Their secreted adhesion proteins can remove interfacial water from contacting surfaces, forming strong bonds through covalent and noncovalent interactions.^[^ ^26^ ^]^ For mussel‐inspired hydrogels, catechol groups play the dominant role in establishing adhesion. However, the uncontrolled oxidized forms of catechol usually lead to weak adhesiveness.^[^ ^28^, ^29^ ^]^ Some special structural designs, such as dome shapes, can also enhance adhesion.^[^ ^30^ ^]^ But the pattern design operation is tremendously complex. Alternatively, the utilization of supramolecular interactions (mainly including hydrogen bonding interactions, hydrophobic interactions, and electrostatic interactions) provides a facile and effective method to achieve appreciable adhesion.^[^ ^31^, ^32^, ^33^ ^]^ Supramolecular bioadhesives are usually composed of permanent polymer networks and dynamic bonds for efficient energy dissipation, thus enabling strong toughness and stretchability.^[^ ^34^, ^35^ ^]^ In contrast, supramolecular adhesives can provide additional functions, such as reversible adhesion, self‐healing, and stimulus responsiveness, which are especially required in some biomedical applications.

It would be of great significance to broaden the application scenarios if a tailored adhesive reveals strong, stable, and adjustable adhesion capability in both dry and wet conditions, but the relevant studies have remained unexplored. The sole supramolecular interactions show strong dry adhesion but might not be sufficient for stable wet adhesion. Previous studies described that light stimulation could induce bonding and self‐assembly in supramolecular polymers to enhance adhesion.^[^ ^36^ ^]^ We conceive that if supramolecular polymers are photocrosslinked in situ on wet surfaces, the weakness of supramolecular interactions would be compensated. Specifically, for in situ polymerized adhesives, the physical entanglement and mechanical interlocking often play a role in tissue adhesion. The adhesive precursor applied to tissue surfaces can penetrate into the matrix and form a firm interpenetrating network upon polymerization,^[^ ^37^, ^38^ ^]^ therefore reinforcing the tight attachment to the wet tissue. We envision that the combination of supramolecular chemistry with in situ polymerization provides a great possibility of developing appreciable dry and wet adhesion.

**Figure 1:**
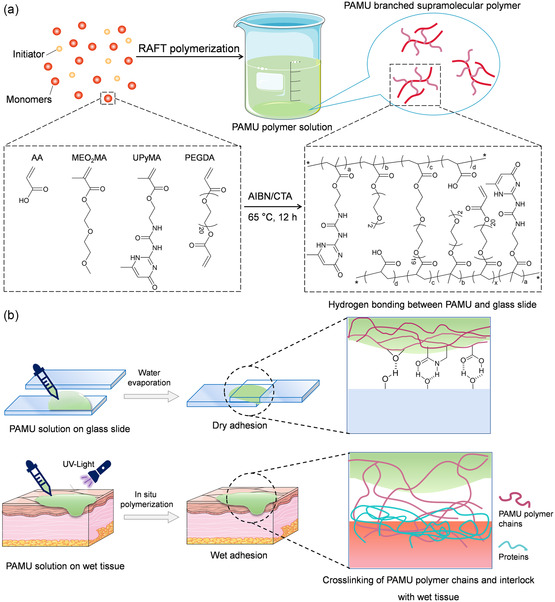
a,b) Schematic illustrations of the synthesis of the branched supramolecular polymer by RAFT polymerization (a) and the dry and wet adhesive application (b).

Here, we propose a dry/wet adhesive based on a supramolecular branched polymer (PAMU). Our synthesis strategy is conducting one‐pot reversible addition‐fragmentation chain transfer polymerization (RAFT) of multiple monomers, including 2‐ureido‐4[1H]‐pyrimidinone (UPy)‐functionalized methacrylate (UPyMA), acrylic acid (AA), and 2‐methyl‐2‐acrylate‐2‐(2‐methoxyethoxy) ethyl ester (MEO_2_MA) and poly(ethylene glycol) diacrylate (PEGDA) (**Figure** **1**). The resulting PAMU solution can achieve strong adhesion to dry solid surfaces after water evaporation. Being photocrosslinked via the polymerization of residual double bonds, the aqueous solution of PAMU polymer turns into a supramolecular hydrogel adhesive. The multiple hydrogen bonds and potential mechanical interlocking endow the PAMU hydrogel with appealing dynamic self‐healing properties as well as stable adhesion to biological tissues. In vitro cytotoxicity and in vivo mouse subcutaneous implantation experiments show the good cytocompatibility and histocompatibility of the PAMU polymer or hydrogel. This study provides a promising approach to develop supramolecular adhesives for versatile adhesion applications from hard substrates to soft biological tissues.

## 2 Results and Discussion

The branched supramolecular PAMU polymer was synthesized by the RAFT polymerization of PEGDA, AA, MEO_2_MA, and UPyMA monomers. The branched polymers PAM (copolymerization of PEGDA, AA, and MEO_2_MA) and PA (copolymerization of PEGDA, AA) were also prepared for comparison. GPC was first used to measure the molecular weight and molecular weight distribution of different polymers, and the results are shown in **Table** **1**.

**Table 1:** Molecular weight and distribution of PA, PAM, and PAMU branched polymers

Sample code	*M*_n_ [Da]	*M*_w_ [Da]	PDI
PA	8,550	21 921	2.564
PAM	12 501	29 182	2.334
PAMU	26 510	59 082	2.229

**Figure 2:**
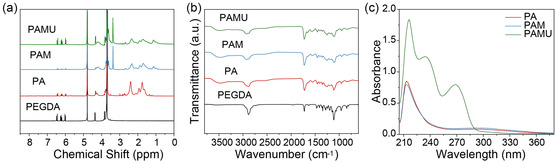
Structural characterization of PAMU and control polymers. a) ^1^H‐NMR spectra of the PAMU, PAM, PA, and PEGDA polymers. b) FTIR spectra of the PAMU, PAM, PA, and PEGDA polymers. c) UV–vis absorption spectra of the PA, PAM, and PAMU polymers.

The ^1^H‐NMR spectra of the branched polymers are shown in **Figure** **2**a. PAMU, PAM, and PA all have three characteristic peaks at 6.0–6.5 ppm, which are double bonds of PEGDA. It is indicated that the three polymers of PA, PAM, and PAMU all have residual double bonds, which could be further crosslinked by UV light. Compared with PA, both PAM and PAMU show characteristic peaks at 1.1 ppm and 3.39 ppm, which are assigned to the side chain methyl group and the terminal methyl group of MEO_2_MA units, respectively. The synthesized polymers were also characterized by Fourier transform infrared (FTIR) spectroscopy (Figure 2b). The FTIR spectra of all synthesized polymers show an absorption band at 1,721 cm^−1^ due to the C=O of poly(ethylene glycol) diacrylate and an absorption band at 2,926 cm^−1^ due to the asymmetric stretching of the methylene group. Peaks are observed at 3,494 and 2,539 cm^−1^ due to the –OH of the carboxyl group of PA, demonstrating the presence of AA and PEGDA incorporated in all polymers. Compared with PA and PAM, the absorption peak of PAMU at 1664 cm^−1^ can assign to the stretching vibration peak of amide C=O, which implies that the polymer was grafted with UPyMA.^[^ ^39^, ^40^ ^]^ UV–vis spectroscopy was further conducted to quantify the incorporated ratio of UPy moieties in the PAMU polymer. As shown in Figure 2c, the aqueous solution of PAMU has an absorption peak at 270 nm, while the PA and PAM solutions have no absorption peak. This indicates that UPyMA was successfully grafted onto the PAMU polymer, and the content of UPyMA in the polymer was quantified by the absorbance of PAMU at 270 nm. It can be obtained from the standard curve equation calculated above that the mass ratio of UPyMA in the PAMU polymer is 5.8%.

**Figure 3:**
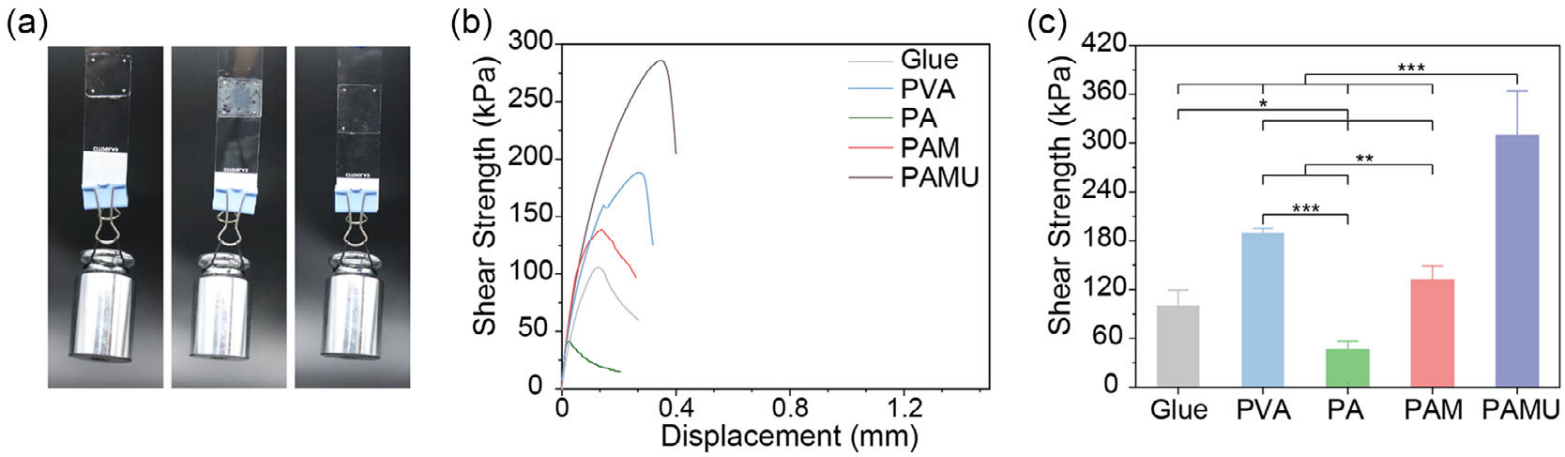
Dry adhesive properties of PAMU and control polymers. a) Photograph of lap shear test setup with 50 g of mass, from left to right: PAMU, PVA, and commercial glue (DeLi 7302, PVA). b) Representative lap shear strength–displacement curves. c) Average lap shear strength of PAMU and different control polymers, including PA, PAM, PVA, and commercial glue. *P < 0.05, **P < 0.01, ***P < 0.001. All data are mean ± standard deviation (SD); *n* = 3 independent samples per group. Statistics were calculated by one‐way ANOVA followed by Tukey's pos*t*‐test.

To test the adhesion properties of PAMU polymers under dry conditions, we applied the PAMU solution, PVA solution, and commercial glue to two slides and observed the adhesion of the glass pieces. As shown in **Figure** **3**a, all samples demonstrate strong adhesion after water evaporation. The shear force from hanging a 500 g weight cannot separate the glass slides. Moreover, the adhesion strength of PAMU, PAM, PA, PVA solution, and commercial glue (DeLi 7302, PVA) is quantitatively characterized by a lap shear test. As shown in Figure 3b,c, the adhesion strength of PAMU with a value of 309.6 ± 54.3 kPa is obviously higher than that of other polymers and commercial glues. This high adhesion strength of the PAMU supramolecular polymer might be attributed to the high cohesive energy and the abundant hydrogen bonds formed between the polymer and glass sides.

**Figure 4:**
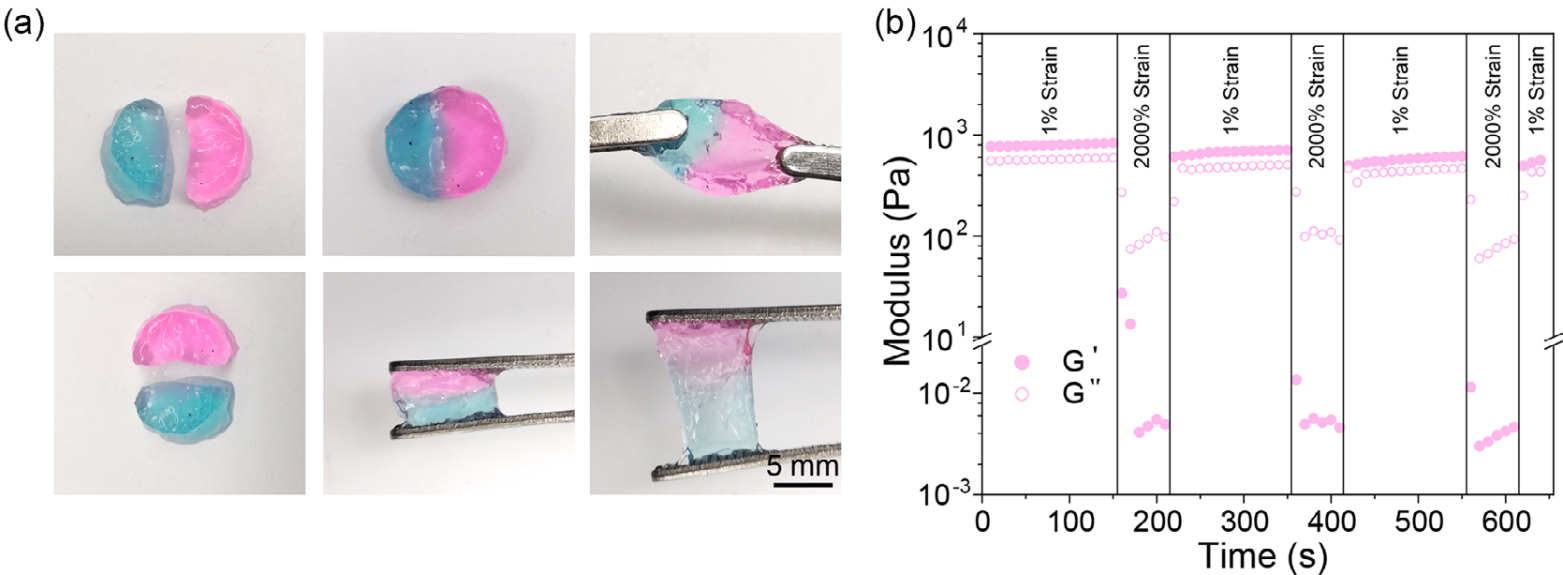
Self‐healing properties of PAMU hydrogel. a) Self‐healing evaluation of PAMU hydrogel. b) Time sweep curve of the PAMU hydrogel under alternative strain cycles.

The prepared polymer solution injected onto the wet tissue surface can in situ cross‐link to form a hydrogel after UV irradiation because of the residual double bonds in polymers. The multiple hydrogen bonding interactions of UPy moieties as well as carboxyl groups endow the PAMU hydrogel with good self‐healing properties. As shown in **Figure** **4**a, after being placed at 37 °C for 2 h, the two hydrogel pieces dyed with rhodamine and methylene blue were completely fused, and the integrated hydrogel could withstand stretching by tweezers. To further evaluate the self‐healing properties of the hydrogel, a continuous step‐strain (1% strain to 2000% strain to 1% strain) scanning procedure is conducted by a rheometric test. As shown in Figure 4b, when the PAMU hydrogel is exposed to 2000% shearing strain, the storage modulus (*G*′) immediately decreases from 870 to 0.004 Pa, which is smaller than the loss moduli (*G*″), indicating that the crosslinking network is destroyed and subsequently the hydrogel turns into a sol state. However, when the strain is reduced to 1%, the hydrogel *G*′ becomes larger and finally recovers to 88% of the initial modulus, which reveals that the broken hydrogel can self‐heal quickly. All these results evidently illustrate that the PAMU hydrogel has the potential to restore its original structure after being damaged and possesses an excellent self‐healing property.

**Figure 5:**
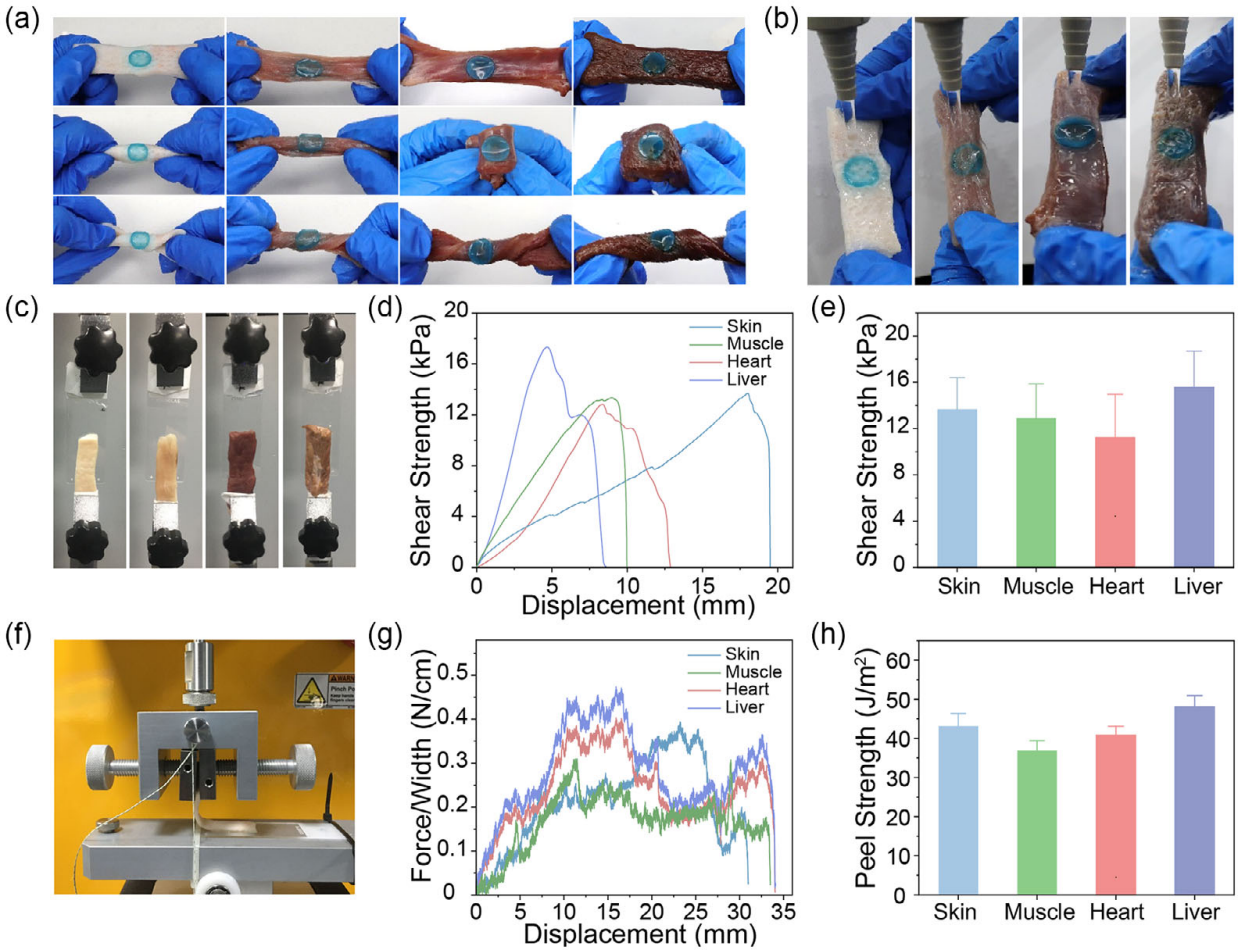
Wet‐adhesion properties of PAMU hydrogel. a) PAMU hydrogel (methylene blue stained) stably adheres to different tissues (from left to right: porcine skin, muscle, heart, and liver) and withstands stretching, bending, and twisting. b) PAMU hydrogel maintains stable adhesion underwater flushing after immersion in normal saline for 30 min. c) Photograph of the lap shear test setup for the adhesion measurement. d) Representative lap shear strength–displacement curve and e) average lap shear strength of PAMU hydrogel on different tissues. f) Photograph of the 90° peel test setup for the adhesion measurement. g) Representative peel strength–displacement curve and h) average peel strength of PAMU hydrogel on different tissues.

We and other groups have demonstrated that the in situ polymerization of macromonomers enables significantly improved wet adhesion to hydrogel or biological tissues.^[^ ^41^, ^42^, ^43^, ^44^ ^]^ The penetration and entanglement of polymer chains facilitate mechanical interlocking with wet tissues, which enhances hydrogel adhesive strength. The adhesion properties of the PAMU hydrogel under wet conditions are demonstrated in **Figure** **5**. The PAMU hydrogel is able to maintain strong adhesion to these tissue surfaces, even during stretching, bending, stretching, twisting, and water flushing (Figure 5a,b). The adhesion strength of the PAMU hydrogel to biological tissues was measured by both lap shear and 90^o^ peel tests (Figure 5c–h). The PAMU prepolymer solution is applied to the surface of the polyacrylamide hydrogel and different tissues, and then light‐initiated polymerization is proceeded to ensure interfacial adhesion. The test results show that the adhesion strength of the PAMU hydrogel to pig skin, muscle, heart, and liver is 13.7, 12.9, 11.2, and 15.5 kPa, respectively (Figure 5e). This adhesion strength of PAMU hydrogel is lower than that of previously reported hydrogel adhesion induced by in situ polymerization, but it is relatively higher compared to catechol‐inspired hydrogel adhesives.^[^ ^43^, ^44^, ^45^, ^46^ ^]^ PAMU hydrogels exhibit excellent peel strengths on various tissues with peel energies of 43.1 ± 3.1, 36.9 ± 2.4, 33.6 ± 2.3, and 48.1 ± 2.8 J m^−2^ (Figure 5h). Overall, the PAMU hydrogel has robust adhesion to most biological soft tissues, and it can serve as a universal binder for other nonadhesive hydrogels to achieve adhesion to biological tissues.

**Figure 6:**
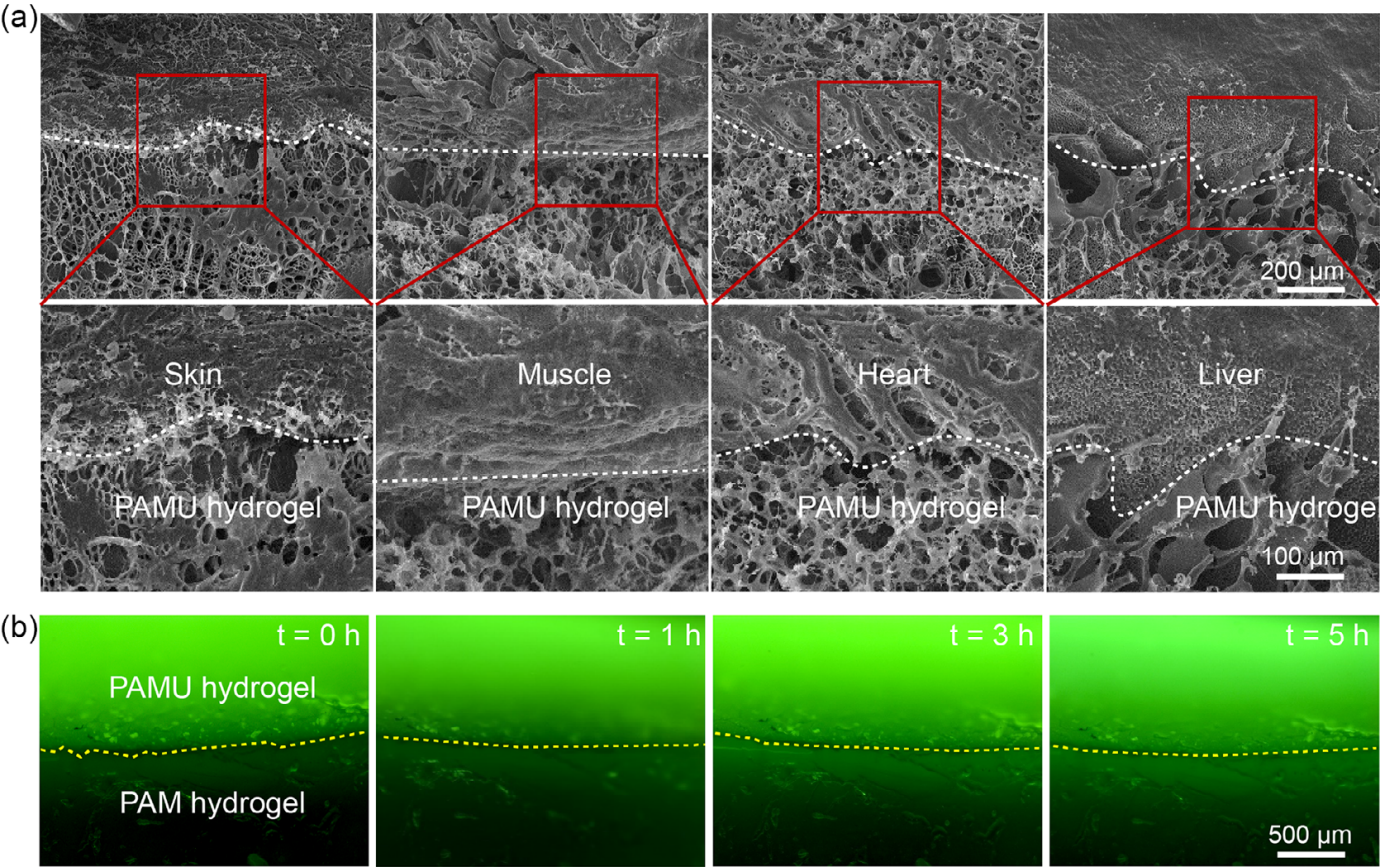
Interfacial observation of PAMU hydrogel adhering to biological tissues and delivering guest molecules to hydrogel substrate. a) SEM images of the PAMU hydrogel adhered to various tissues. The white dotted line marks the contact interface between the hydrogel and tissues. b) Fluorescence images of FITC molecules diffusing from the PAMU hydrogel to the polyacrylamide hydrogel.

SEM imaging is used to observe the adhesion interface of the PAMU hydrogel with different tissues to further illustrate the tight adhesion of the PAMU hydrogel from a microscopic point of view. As shown in **Figure** **6**a, the lyophilized biological tissues (pig skin, muscle, heart, and liver) reveal dense structures, while the PAMU hydrogel has macroporous structures, but there is no obvious gap between the PAMU hydrogel and tissues, indicating the tight adhesion and integration. Fluorescein isothiocyanate (FITC) fluorescein is incorporated into the PAMU hydrogel to evaluate its permeability. The fluorescence images of PAMU combined with polyacrylamide incubated for different times are shown in Figure 6b. Initially, fluorescence only exists in the PAMU hydrogel, while the polyacrylamide hydrogel has almost no fluorescence. However, with increasing incubation time, sufficient penetration could allow FITC molecules to diffuse from the PAMU hydrogel to the polyacrylamide hydrogel. The fluorescence intensity and the thickness of the fluorescent layer in the polyacrylamide hydrogel gradually become stronger and thicker. It could be anticipated that through the adhesion and permeability properties of PAMU hydrogels, a hydrogel drug‐loading platform can probably be constructed. The PAMU hydrogel could be firmly attached to the target tissue, and the encapsulated drug could gradually penetrate into the tissue to achieve the targeted effect and sustainable drug release.

**Figure 7:**
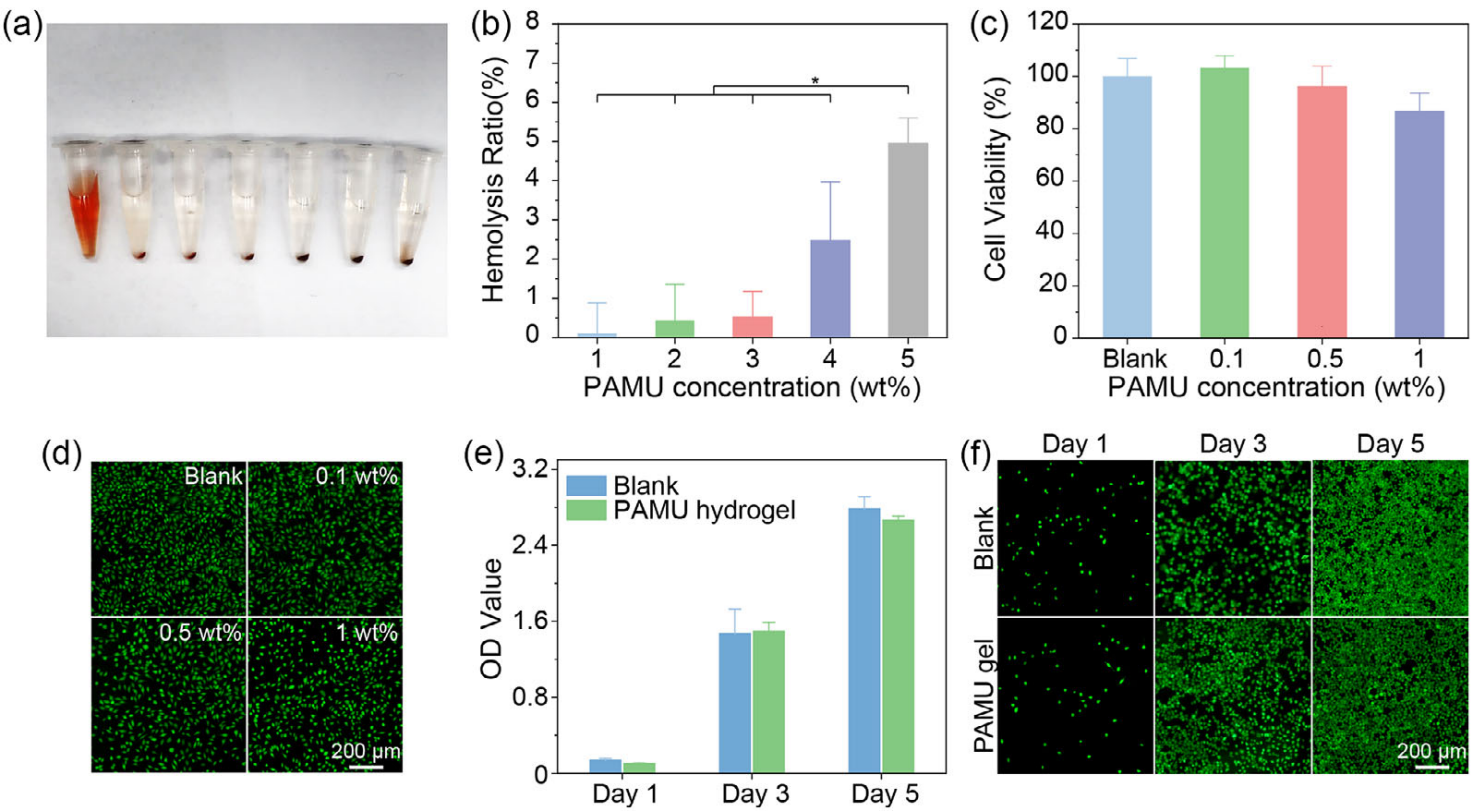
In vitro biocompatibility characterization of the PAMU polymer and hydrogel. a) Photograph of hemolysis tests, from left to right: positive deionized water group, negative PBS group, PAMU polymer solution groups with concentrations of 1.0, 2.0, 3.0, 4.0, and 5.0 wt%. b) Hemolysis rate of PAMU polymer solutions. *P < 0.05. c) Cell viability and d) live/dead staining images of L929 cells in PAMU polymer extract at various concentrations. e) OD values of L929 cells cultured with PAMU hydrogel extracts. f) Live and dead staining images of L929 cells cultured with PAMU hydrogel extract after 1, 3, and 5 days. All data are mean ± SD; *n* = 3 independent samples per group. Statistics were calculated by one‐way ANOVA followed by Tukey's pos*t*‐test.

Hydrogel adhesives should have good blood compatibility for use as hemostatic agents or wound dressings. We tested the hemolysis rate of PAMU polymer solutions at concentrations from 1 to 5 mg mL^−1^. **Figure** **7**a shows the hemolysis results of the PAMU groups, negative PBS group, and positive deionized water group after centrifugation. The PAMU polymer groups are similar to the PBS control group without obvious hemolysis, while the positive group has severe hemolysis. From the quantitative data analysis of the OD value in Figure 7b, it can be seen that the hemolysis rate of all PAMU groups is lower than the critical and safe hemolytic ratio for biomaterials (5%) according to ISO/TR 7406. The above results indicate that the PAMU polymer has excellent blood compatibility. L929 cells are cocultured with different concentrations of PAMU polymer for 24 h, and then cell viability is tested using CCK‐8 and live/dead staining to assess the cytotoxicity of the PAMU polymer. In Figure 7c, cells incubated with different concentrations of PAMU polymer extracts for 24 h show good viability (>85%). In addition, the morphology and proliferation of the cells are observed after live/dead staining. From the fluorescence images in Figure 7d, it can be seen that the cells cultured with different concentrations of PAMU polymer extract showed normal cell spreading morphology, high viability, and no significant difference from that in normal culture conditions.

To further verify the cytocompatibility of the PAMU hydrogel, the extract solution of the PAMU hydrogel (20 mg mL^−1^) is used for cell culture. The OD value of cells cultured with the PAMU hydrogel increases significantly with the extension of culture time (Figure 7e). There is no significant difference with the blank group, which reveals that the PAMU hydrogel does not affect the natural proliferation of cells. In contrast, the laser confocal images show that there are almost no dead cells after 5 days of PAMU hydrogel leaching solution (Figure 7f). Therefore, all of these toxicity assays demonstrate the high biosafety and biocompatibility of the PAMU polymer and hydrogel.

**Figure 8:**
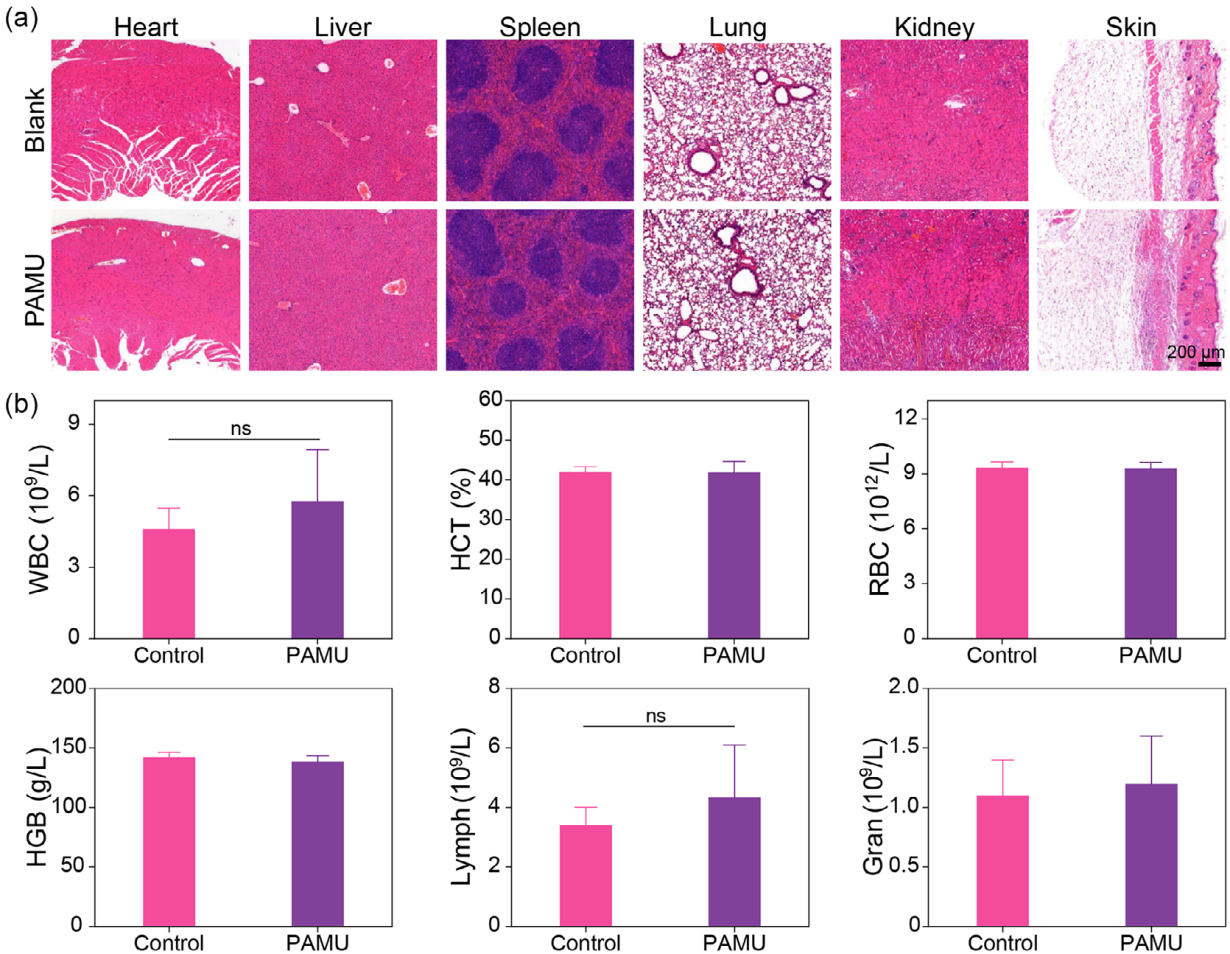
In vivo biosafety evaluation. a) H&E staining of heart, liver, spleen, lung, kidney, and skin tissue after subcutaneous implantation of PAMU hydrogel in the back of mice for 10 days. b) Blood biochemical analysis of white blood cells (WBC), hematocrit (HCT), red blood cells (RBC), hemoglobin (HGB), lymph (lymphocytes), and gran (granulocytes) after subcutaneous implantation of PAMU hydrogel for 10 days. All data are mean ± SD; *n* = 3 independent samples per group. Statistics were calculated by one‐way ANOVA followed by Tukey's pos*t*‐test.

To further investigate the in vivo biocompatibility, the PAMU hydrogel was implanted subcutaneously on the back of mice, and 10 days postimplantation, blood and major organs were collected for blood biochemical examination and hematoxylin and eosin (H&E) staining. The H&E staining results (**Figure** **8**a) show no obvious tissue inflammation or pathological changes in any of the major organs following hydrogel administration for 10 days. Hematological parameters in serum are studied and compared, including white blood cells (WBCs), red blood cells (RBCs), alanine aminotransferase (ALT), aspartate aminotransferase (AST), hematocrit (HCT), hemoglobin (HGB) and other blood biochemical parameters (Figure 8b). No significant changes are found between the experimental group and the healthy rats. The above results show that the PAMU hydrogel has good in vivo biocompatibility and might potentially serve as a surgical sealant for soft‐tissue wound sealing and healing.

## 3 Conclusion

We have developed a branched supramolecular polymer synthesized by RAFT polymerization to achieve both dry and wet adhesion. The supramolecular polymer solution demonstrates high dry adhesion strength. After photoinitiated in situ polymerization, the PAMU polymer can be further crosslinked to form a supramolecular hydrogel. The multiple hydrogen bonds of UPy and carboxyl moieties endow the supramolecular hydrogel with dynamic self‐healing properties. The mechanical interlocking between polymer and tissue enables its promising wet adhesion properties to biological tissues. The in vitro cytotoxicity, and in vivo biosafety evaluation demonstrate the good biocompatibility of the supramolecular polymer or hydrogel. Taken together, this work paves a new way for developing adhesives with tunable dry and wet adhesion for versatile applications.

## 4 Experimental Section

4.1

4.1.1

##### Materials

Anhydrous dimethyl sulfoxide (DMSO), poly(ethylene glycol) diacrylate (PEGDA), acrylic acid (AA), azobisisobutyronitrile (AIBN), *N,N*‐dimethylformamide (DMF), and 2‐methyl‐2‐acrylic acid‐2‐(2‐methoxyethoxy)ethyl ester (MEO_2_MA) were purchased from Aladdin. UPyMA was prepared according to previous literature.^[^ ^47^, ^48^, ^49^ ^]^ 4‐Cyano‐4‐(phenylcarbonothioylthio)pentanoic acid, photoinitiator Irgacure 2959 (I2959), and gelatin (type A, from porcine skin) were provided by Sigma‐Aldrich. Acrylamide (AM), calcium chloride (CaCl_2_, 96%), and FITC were purchased from Macklin. Dulbecco's modified Eagle's medium (DMEM), fetal bovine serum (FBS), phosphate‐buffered saline (PBS, pH = 7.4), pancreatin, penicillin–streptomycin solution, and live‐dead staining solution were purchased from Life Technologies (Gibco, USA). The Cell Counting Kit‐8 was purchased from DOJINDO (Japan). Anticoagulated rabbit blood (anticoagulant with sodium citrate) obtained from Guangzhou Hongquan Biotechnology was used for the hemolysis test. Mouse fibroblastic cells (L929) were provided by The Global Bioresource Center (ATCC). Paraformaldehyde (PFA, 4%) was purchased from Beijing Leagene Biotechnology. All the other reagents were of analytical grade and used as received.

##### Synthesis of Supramolecular Branched Polymers

PAMU was prepared by RAFT polymerization. A total of 1.0 g PEGDA, 2.16 g AA, 0.28 g UPyMA, 0.028 g 4‐cyano‐4‐(phenylcarbonothioylthio)pentanoic acid, 470 μL of MEO_2_MA, and 95 mL of DMF were mixed together in a flask with stirring at room temperature, while nitrogen was continuously introduced to exclude dissolved oxygen for 45 min. Then, 0.0328 g AIBN dissolved in 5 mL of DMF was added into the flask. The flask was quenched with ice water and exposed to air to stop the reaction after 12 h in the oil bath at 65 °C. Afterward, to purify the product, a great amount of deionized water was used for the dialysis (molecular weight cut‐off: 3500 Da). Finally, the PAMU was freeze‐dried and stored at −20 °C before use. The control polymers without adding UPyMA and neither UPyMA nor MEO_2_MA were also prepared, which were named PAM and PA, respectively. The preparation method was the same as that of PAMU.

##### Chemical Structural Characterization of PAMU Polymer

To investigate the chemical structure of PAMU, ^1^ H‐NMR (AVANCE III HD 400, Bruker, Germany) and FTIR (VERTEX 33, Bruker, Germany) were used. PAMU polymer was dissolved in CDCl_3_ and D_2_O, respectively, with a concentration of 10 mg mL^−1^. ^1^ H‐NMR characterization was carried out at 400 MHz. FTIR spectroscopy was used to collect infrared spectra in total reflection mode.

To determine the UPyMA content in PAMU, UV–vis spectrometry (UV‐2600, Shimadzu, Japan) was used to measure the absorbance of PA, PAM, and PAMU at 270 nm. 0.01 m NaOH solution was applied to facilitate dissolving UPyMA. UPyMA solutions with concentrations of 5, 10, 20, 25, 30, and 40 μg mL^−1^ were used to fit the standard curve.

The absolute molecular mass and molecular weight distribution of the polymers were measured by using gel permeation chromatography (GPC, e2695, Waters, USA) equipped with a refractive index and double‐angle light scattering detectors. The mobile phase was water and 20% v/v methanol, the flow rate was 1 mL min^−1^, and the injection volume was 50 μL.

##### Evaluation of the Self‐Healing Properties of PAMU Hydrogel

The macroscopic self‐healing performance of the PAMU hydrogel was evaluated as follows. The hydrogel was cut into two pieces and dyed with methylene blue and rhodamine dye, respectively. The hydrogel pieces were in contact with each other for 2 h. The modulus change of the PAMU hydrogel before and after healing was analyzed by rheological tests (MCR 302, Anton Paar, Austria). Amplitude oscillatory strains at alternating high (2000%) and low (1%) values were applied in the sweeping tests. The angular frequency was 10 rad s^−1^, and the temperature was 25 °C. The PAMU hydrogel was destroyed by applying a strain of 2000% for 50 s and recovered by applying a strain of 1% for 150 s.

##### Evaluation of the Adhesive Properties of PAMU Polymer and Hydrogel

The adhesive property of the PAMU polymer was assessed by lap shear test using a universal testing machine (Instron 5967, USA) with a tensile speed of 5 mm min^−1^. The PAMU, PA, PAM polymer solution (50 wt%) was placed on the opposite position of two glass slides (25 mm × 20 mm) and dried in a 60 °C oven overnight before the test. PVA and commercial glue were also tested for comparison.

The adhesive property of the PAMU hydrogel was assessed by both lap shear and 90° peel tests. For the lap shear test, the PAMU polymer solution was injected on the opposite position of the porcine tissue surface, and a glass slide coating with freshly prepared polyacrylamide hydrogel acted with the tissue under a 50 g weight. The polymer solution (20 wt%, 75 μL) was gelled under an ultraviolet lamp for 2 min (power density: 50 mW cm^−2^, wavelength: 365 nm). The shear stress of the PAMU hydrogel was tested at a tensile rate of 10 mm min^−1^ using a universal testing machine (Instron 5967, USA). For the 90^o^ peel test, PAMU polymer solution (25 wt%, 50 μL) was injected onto the surface of the porcine tissue (skin, muscle, heart, and liver) with a coating area of 20 mm × 10 mm. Then, the glass slide coated with gelatin was placed on the polymer solution. The polymer solution was gelled under an ultraviolet lamp for 2 min. The peel stress was tested by a texture analyzer (CT3 4500, Brookfield, USA) at a tensile rate of 0.5 mm s^−1^.

##### Characterization of Permeability Properties of PAMU Hydrogel

A polyacrylamide hydrogel was first prepared, and then the PAMU prepolymer solution containing FITC solution was added to the surface of the polyacrylamide hydrogel and cured by UV irradiation. After incubation for 1, 3, and 5 h, the infiltration of fluorescence at the interface between the PAMU hydrogel and polyacrylamide hydrogel was observed using an inverted fluorescence microscope.

##### In Vitro Biocompatibility Characterization of the PAMU Hydrogel

The in vitro hemolysis of the PAMU hydrogel was evaluated by fresh rabbit whole blood (sodium citrate anticoagulation). Red blood cells were separated from serum by centrifugation at 3000 rpm for 10 min. PAMU hydrogels of different masses (1–5 mg) were added to 1 mL of PBS and mixed with 200 μL of 2% v/v purified red blood cell suspension after complete dissolution. The resulting mixed solution was incubated at 37 °C for 2 h and then centrifuged at 3000 rpm for 10 min. The supernatant (100 μL) was pipetted into a 96‐well plate, and the absorbance at 540 nm was measured using a microplate reader. Deionized water (1 mL) and PBS (1 mL) mixed with 200 μL of 2% v/v red blood cell suspension were used as positive and negative controls, respectively. The hemolysis rate was calculated using the following formula

(1) $$\text{Hemolysis}\left(\%\right)=\left({\text{OD}}_{\text{sam}}-{\text{OD}}_{\text{neg}}\right)/\left({\text{OD}}_{\text{pos}}-{\text{OD}}_{\text{neg}}\right)\times 100\%$$

OD_sam_ is the absorbance of the sample, OD_neg_ is the absorbance of the negative control group, and OD_pos_ is the absorbance of the positive control group.

Mouse fibroblast L929 cells were used to evaluate the biocompatibility of the PAMU polymer and hydrogel. Sterilized PAMU polymer was soaked in 10 mL DMEM containing 10% v/v fetal bovine serum (FBS) and 1% v/v penicillin–streptomycin to prepare 0.1, 0.5, and 1 wt% solutions and filtered through filter membranes (0.22 μm). Next, L929 cells were seeded into 48‐well plates at a density of 2 × 10^4^ cells mL^−1^ and cultured in the PAMU solutions for 24 h. Then, the culture medium was aspirated, and the cells were washed with PBS. The optical density (OD) value was measured at 450 nm using a microplate reader to test cell viability. Subsequently, 200 μL of the live and dead staining kit was introduced into each well and incubated for 30 min at 37 °C. After staining, the cells were observed by laser scanning confocal microscopy. The biocompatibility of the PAMU hydrogel was evaluated according to the same protocol.

##### In Vivo Histocompatibility Characterization of the PAMU Hydrogel

Animal experiments were carried out in accordance with the “Guidelines for the Care and Use of Laboratory Animals of South China University of Technology” and approved by the University Animal Ethics Committee. The dorsal skin of female BALB/c mice (6–7 weeks old) was incised and sutured after subcutaneous implantation of the PAMU hydrogel (*n* = 3). Mice were sacrificed 10 days later, and major organs (heart, liver, spleen, lung, kidney, and skin) were collected for H&E staining analysis. Mouse blood was collected for routine blood tests (EDTAK2 as an anticoagulant) to assess the toxicity of PAMU hydrogels. Normal mice without any treatment were used as controls.

##### Statistical Analysis

All statistical data are presented as the mean ± SD. The statistical significance of differences was determined using analysis of variance (one‐way ANOVA with Tukey's test). Statistical differences were defined as *P < 0.05, **P < 0.01, ***P < 0.001, and *P* < 0.05 was considered significant.

## Conflict of Interest

The authors declare no conflict of interest.

## Data Availability

The data that support the findings of this study are available from the corresponding author upon reasonable request.
